# Supplementary material for: Excited-state vibration-polariton transitions and dynamics in nitroprusside
Source: Nat Commun. 2021 Jan 11;12:214. doi: 10.1038/s41467-020-20535-z (PMC7801531; doi:10.1038/s41467-020-20535-z)
Supplement: Supplementary file 1 — Supporting Information [file 41467_2020_20535_MOESM1_ESM.pdf]

Supplementary Information for

Excited-State Vibration-Polariton Transitions and Dynamics in Nitroprusside

Andrea B. Grafton,[1] Adam D. Dunkelberger,[2] Blake S. Simpkins,[2] Johan F. Triana,[3] Federico Hernandez,[4] Felipe Herrera,[3] Jeffrey C. Owrutsky[2]

[1]National Research Council Post-Doctoral Scholar, Washington, DC, USA

[2]Chemistry Division, Naval Research Laboratory, Washington, DC, USA

[3]Department of Physics, Universidad de Santiago de Chile, Santiago, Chile

[4]Department of Chemistry, School of Biological and Chemical Sciences, Queen Mary University of London, London, UK

Corresponding author email: jeff.owrutsky@nrl.navy.mil

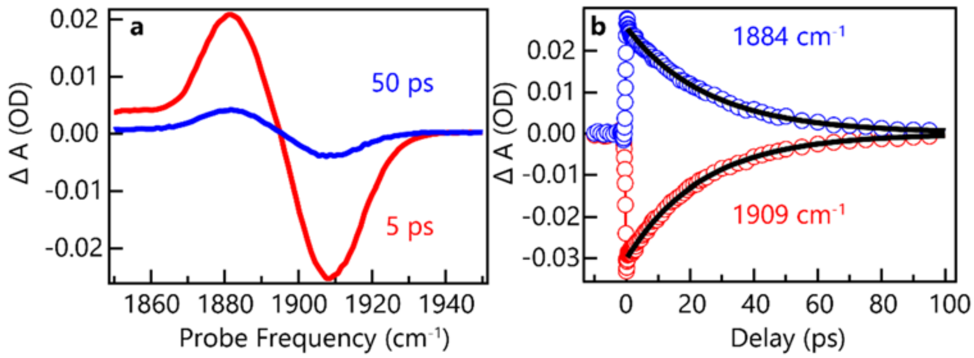

Supplementary Figure 1. Transient absorption and dynamics spectra of free-space nitroprusside. a) Transient infrared spectra of SNP in methanol in free space measured 5 ps (red) and 50 ps (blue) after excitation. **b**) Kinetic traces measured at the peak of the ground-state bleach (1909 cm^{-1}, red) and the excited-state absorption (1884 cm^{-1}, blue). Solid black lines are fits to an exponential decay. The fit yields a time constant of 24 ps for the ground-state bleach and 25 ps for the excited-state absorption.

Supplementary Note 1. **Analytical Modeling of Reservoir Modified Transmission.**

We have previously reported our use of a standard method for calculating the transmission through a Fabry-Pérot cavity containing an absorbing medium[1-8]. Below, we briefly describe that model and report the values of the parameters we used for SNP in methanol. The transmission through the cavity given by Equation S1:

$$T_{\text{cav}}(\bar{\nu}) = \frac{T^2 e^{-\alpha L}}{1+R^2 e^{-2\alpha L} - 2Re^{-\alpha L}\cos(4\pi n L\bar{\nu}+2\varphi)} \tag{S1}$$

where, T and R are the transmissivity and reflectivity of the mirrors, taken to be 8% and 92% across the wavelength range of interest, L is the length of the cavity, φ accounts for a phase shift as light reflects from the mirror, and n and α are the frequency-dependent index of refraction and absorption per unit length of the material within the cavity, respectively. To generate n and α, we first describe the dielectric function of SNP in methanol solution in terms of a background real index n_{bg} and sum of two Lorentzian oscillators given by the equations:

$$\varepsilon_1 = n_{\text{bg}}{}^2 + \frac{A_{01}(\nu_{01}{}^2-\nu^2)}{(\nu_{01}{}^2-\nu^2)^2+(\Gamma_{01}\nu)^2} + \frac{A_{12}(\nu_{12}{}^2-\nu^2)}{(\nu_{12}{}^2-\nu^2)^2+(\Gamma_{12}\nu)^2} \tag{S2}$$

$$\varepsilon_2 = \frac{A_{01}\Gamma_{01}\nu}{(\nu_{01}{}^2-\nu^2)^2+(\Gamma_{01}\nu)^2} + \frac{A_{12}\Gamma_{12}\nu}{(\nu_{12}{}^2-\nu^2)^2+(\Gamma_{12}\nu)^2} \tag{S3}$$

Here, we choose values for the fundamental and excited state center frequencies and linewidths to be ν_{01} = 1906 cm^{-1}, ν_{12} = 1883 cm^{-1}, Γ_{01} = 8.6 and Γ_{12} = 21 cm^{-1} to match values for the NO stretch in SNP.[9,10] Specifically, these values correspond to the homogeneous linewidth of the strongly coupled ν_{01} transition and the inhomogeneously broadened width of the excited ν_{12} state since it's in the weak coupling cavity-enhanced absorption regime. We vary the amplitude terms A, ($A_{01} = N_0 \bullet \mu_{01}$; $A_{12} = N_1 \bullet \mu_{12}$) which encompass the transition intensity and population of the absorber, to account for depopulation of the ground state and population of the first excited state. We then use the following expressions to calculate n and α:

$$n = \sqrt{\frac{\varepsilon_1+\sqrt{\varepsilon_1{}^2+\varepsilon_2{}^2}}{2}} \tag{S4}$$

$$\alpha = 4\pi\nu k = 4\pi\nu\sqrt{\frac{-\varepsilon_1+\sqrt{\varepsilon_1{}^2+\varepsilon_2{}^2}}{2}} \tag{S5}$$

Finally, we calculate transmission spectra for the unpumped (N_0 = 1, N_1 = 0) and excited systems (i.e., population moved from N_0 to N_1), subtract the two, and normalize by the ground state transmission to arrive at calculated transient spectra. In all cases, the analytical model predicts a response close to ν_{01}, but the

experimental intensity of transmitted light is below the detection limit of our instrument and we cannot measure a transient signal.

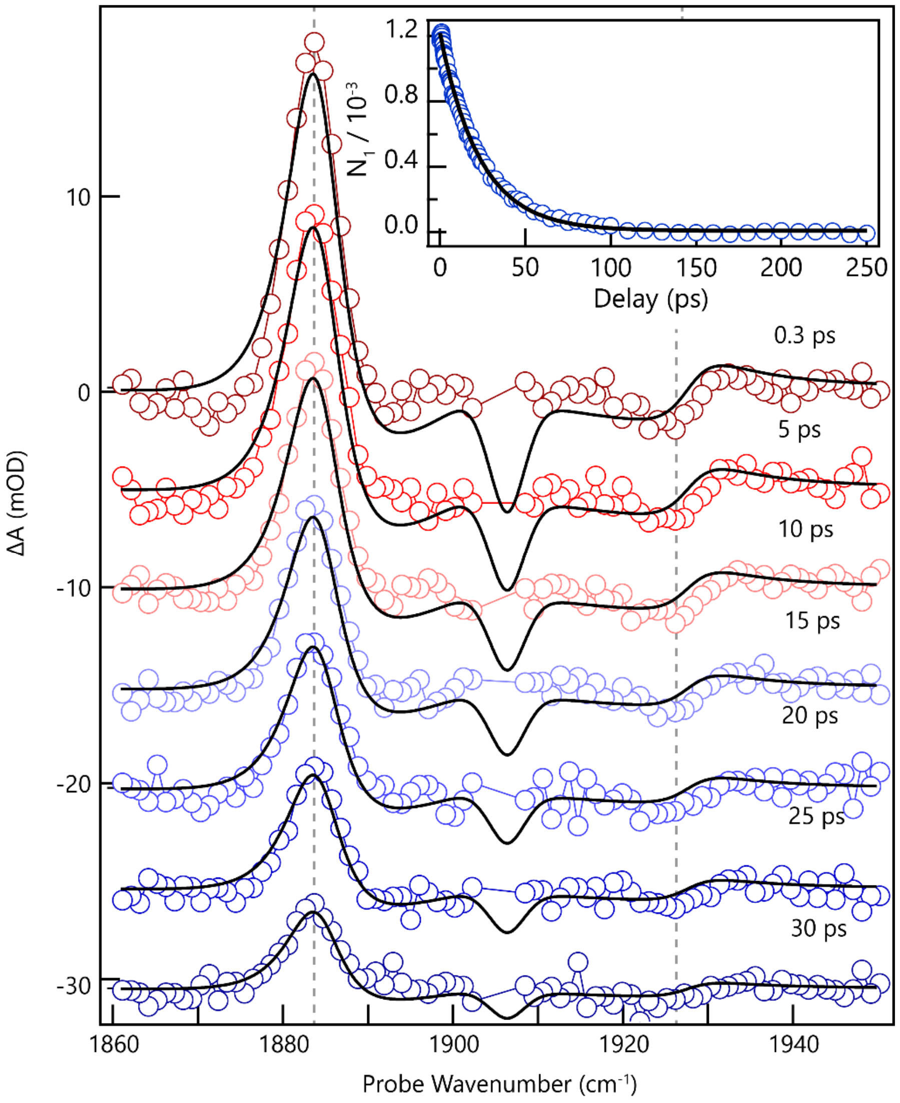

Supplementary Figure 2. Pump-probe spectroscopy of cavity-coupled SNP. Open circles are experimental data obtained after excitation with the Reservoir-Pump mask described in the main text. The data are reported in terms of $-\log(T/T_0)$. Each color corresponds to a different delay time between 0.3 (red) and 30 ps (blue). Solid black lines are fits to the analytical expression for cavity transmission with fractional population, N_1, the only fit parameter; the fit yields N_1 = 0.09%. The gray vertical dash lines indicate frequencies 1883 cm^{-1} and 1926 cm^{-1}.The inset shows the extracted data with exponential fit for excited-state decay yielding a relaxation time constant of $\tau = 23.9$ ps.

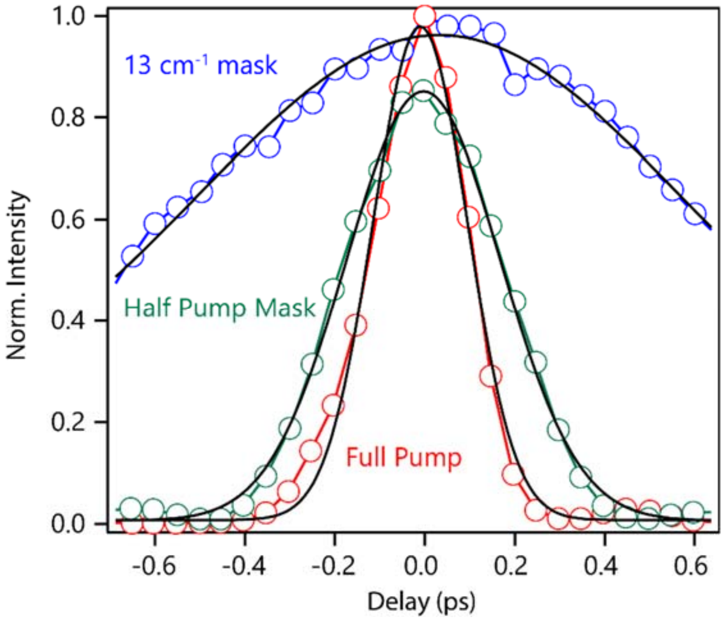

Supplementary Figure 3. Cross correlations of the pump and probe pulses. Open circles are cross correlation data obtained with the Full Pump (red), a representative Half Pump mask (green), and the 13 cm^{-1} mask (blue) used to obtain the Reservoir-Pump spectra in the main text. Black lines are fits to a Gaussian pulse. The full-widths at half-maximum of the Gaussian fits are 0.25 ps for the Full Pump, 0.4 ps for the Half Pump, and 1.3 ps for the 13 cm^{-1} Reservoir-Pump mask.

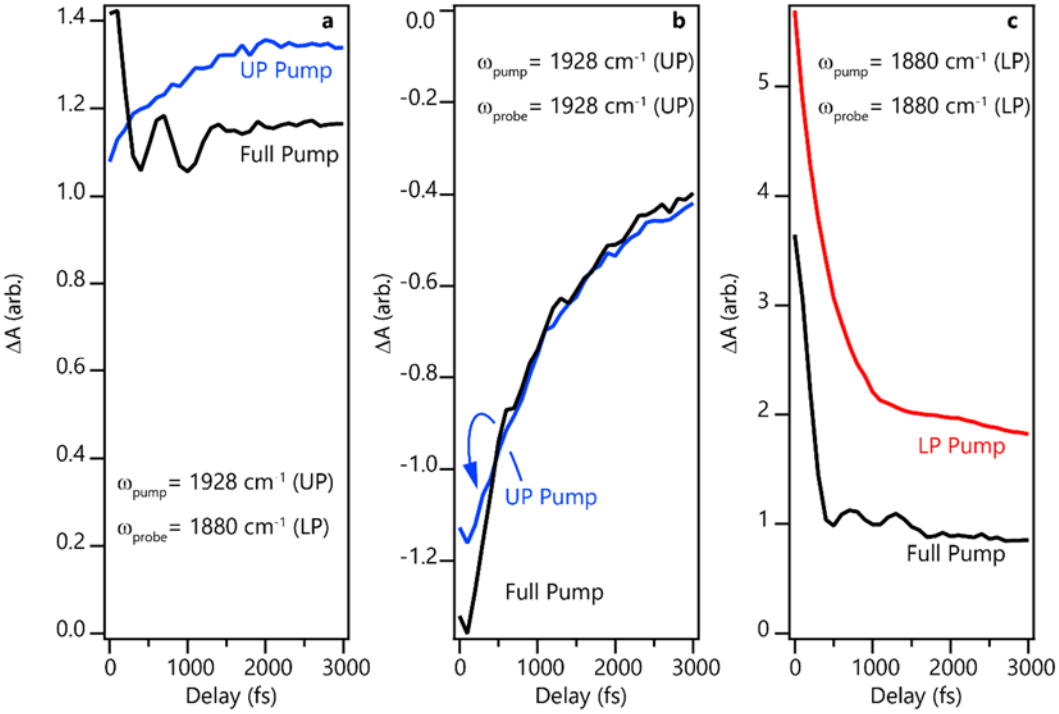

Supplementary Figure 4. Kinetic traces at early time delays. Each panel shows kinetic traces obtained with by ω_{pump} and ω_{probe} with either full pump (black) or half pump (colored) excitation**. a)** ω_{pump} = 1928 cm^{-1} (UP) and ω_{probe} = 1880 cm^{-1} (LP). **b)** ω_{pump} = 1928 cm^{-1} (UP) and ω_{probe} = 1928 cm^{-1} (UP). **c)** ω_{pump} = 1880 cm^{-1} (LP) and ω_{probe} = 1880 cm^{-1} (LP). In all cases, the oscillations are suppressed under half-pump excitation, as described in the main text.

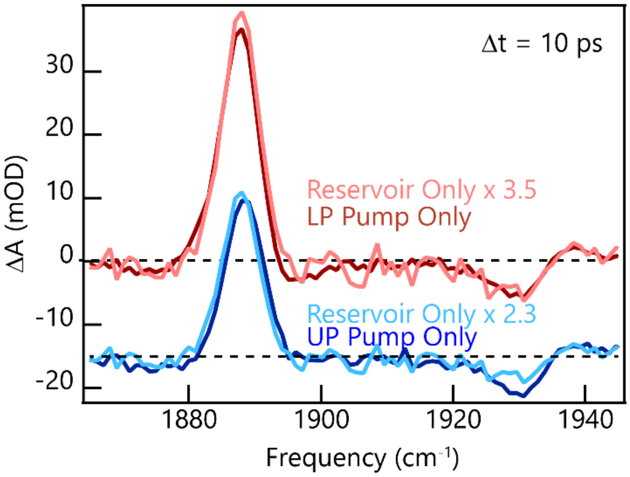

Supplementary Figure 5. Pump-probe spectra obtained with spectrally filtered pump pulses. The dark red trace is the transient spectrum recorded 10 ps after exciting with the LP Pump mask and the light red trace is the spectrum recorded 10 ps after exciting with the Reservoir-Pump mask then multiplied by

3.5, as described in the main text. The blue traces (offset for clarity) are the corresponding spectra for UP Pump mask excitation, for which the scaling factor is 2.3.

Supplementary Note 2. Theoretical Methods and Results

Molecular System Description. We use the results of the free-space transient experiments to determine the transition energies E_{01} = 1909 cm^{-1} and E_{12} = 1884 cm^{-1}. We estimate the transition dipole moments of the relevant transitions by converting the integrated molar absorption coefficients to transition dipole moments with literature methods. For example, the integrated absorption coefficient for the v = 0 to 1 transition is 35,000 M^{-1} cm^{-2}, or 3280 cm^{-2} atm^{-1} [9-11]. We then use the relation,

$$\left|\langle d_{\mathrm{n,n'}}\rangle\right|^2 = {S_v^0 q_v}\Big/{(11.183\nu)} \qquad \text{(S6)}$$

where $d_{\mathrm{n,n'}}$ is the transition dipole moment in Debye, S_v^0 is the integrated absorption coefficient (in cm^{-2} atm^{-1} (base e; converted from the solution base 10 value in M^{-1} cm^{-2}), q_v is the vibrational partition function, which is close to 1 because there is no discernable hot band transition resolved around NO fundamental, and ν is the transition frequency (in cm^{-1}).[12] Absorption spectra were measured in H_2O to provide enough solubility and transmission to observe the overtone absorption simultaneously with the fundamental in FTIR spectra; the integrated band intensity for the overtone is 55 times weaker. The integrated excited state absorption in transient spectra for the NO band (e.g., in Supplementary Fig. 1a above) outside the cavity is nearly the same as for the ground state bleach. Since the population difference for the ground state bleach is twice that for the excited state absorption, then S_v for the excited state is twice the value for the fundamental.

Transition [n',n]	Frequency [cm^{-1}]	S_v [M^{-1} cm^{-2}]	S_v [cm^{-2}atm^{-1}]	$d_{\mathrm{n,n'}}$ [Debye]
1-0, fundamental	1935	35000	3276.6	0.39
2-1, excited state	1907	70000	6553.3	0.55
NO overtone	3851	630	59.0	0.037

Supplementary Table 1. NO band transition frequencies, integrated band strengths and transition moments. Parameters used to determine the dipole moment function for SNP.

Morse Oscillator Model for N-O Stretching Mode. Based on the available experimental data, we model the NO stretching mode of SNP as a Morse oscillator that is weakly coupled to other normal modes. The Morse potential has the form,

$$V(q) = D_e\left(1 - e^{-a(q-5.0)}\right)^2 - D_e. \tag{S7}$$

The reduced mass of the NO stretching mode is 14.75 AMU, obtained from a normal mode calculation on an optimized geometry of SNP at the CAM-B3LYP/6- 311++G** level of theory.[13]. The polarizable continuum model (PCM) tuned with the dielectric constant of methanol (ε = 32.613) is included to account for the solvent electrostatic interactions.

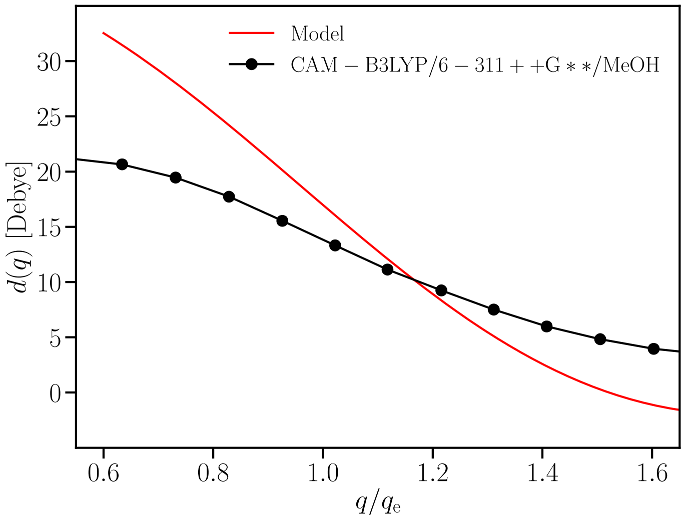

Supplementary Figure 6. **Calculated dipole functions.** Model electric dipole function $d(q)$ (red line) and *ab-initio* DFT dipole function along the NO stretching mode coordinate (circles).

By comparing the exact expression for the Morse vibrational energies $E(v)$ with the Dunham expansion up to second order in the vibrational quantum number v, the vibrational energy spectrum is given by [1]

$$E(\nu) = -D_e + a\sqrt{\frac{2D_e}{\mu}}\left(\nu + \frac{1}{2}\right) - \frac{a^2}{2\mu}\left(\nu + \frac{1}{2}\right)^2 = -D_e + \omega_e\left(\nu + \frac{1}{2}\right) - \omega_e\chi_e\left(\nu + \frac{1}{2}\right)^2 \tag{S8}$$

Using the experimental values for the 1⟵0 and 2⟵1 transitions (see Supplementary Fig. 1, note above), we obtain ω_e = 1934 cm^{-1} and $\omega_e\chi_e$ = 12.5 cm^{-1}. These frequencies correspond to the Morse parameters a = 1.75 a.u and D_e = 0.34 a.u., for the reduced mass obtained with DFT as above. Calculations were performed using the parameters in Supplementary Table 2. We compute the Morse vibrational energies and eigenstates using the Fourier Grid Hamiltonian method[14], considering N = 601 grid points over the interval [3.5, 9.5] a.u along the nuclear coordinate. The corresponding energy parameters and transition energies are given in Supplementary Table 2.

	Theory [cm^{-1}]	Experiment [cm^{-1}]	Difference [cm^{-1}]
$\omega_e\chi_e$	12.8	12.5	0.3
ω_e	1939	1934	5
E_{10}	1913	1909	4
E_{21}	1888	1884	4

Supplementary Table 2. **Morse potential parameters.** Theoretical and experimental energy values ω_e and $\omega_e\chi_e$ of a Morse potential and the vibrational transition energies E_{10} and E_{21}**.** In the Morse model, we set a = 1.77 a.u., D_e = 0.3345 a.u. and μ = 14.73 AMU.

In the Born-Oppenheimer approximation, the contribution of the electron charge distribution to the molecular dipole moment can be represented by function $d(q)$ that depends parametrically on the nuclear coordinates. This dipole function for the NO stretching mode in SNP can in principle be obtained from *ab-initio* calculations. However, we prefer to use a general functional form that can describe the physical behavior of a broad range of molecular species[15,16], which reads

$$d(q) = d_0(q - c_0)e^{-\frac{(q-c_1)^2}{2\sigma^2}} + d_{\mathrm{p}}. \tag{S9}$$

The set of dipole function parameters are chosen to reproduce the transition dipole moments of SNP in methanol, as obtained from experiments. Table 3 shows the values of the experimental and simulated transition dipole moments. Supplementary Fig. 6 shows the model dipole moment function as well as an *ab-initio* (DFT) dipole function along the NO stretching normal mode coordinate, obtained with CAM-B3LYP/6-311++G**/methanol level of theory. Although the two dipole moments curves are not equal, they both predict that the maximum dipole moment occurs at bond distances shorter than equilibrium. In other words, NO in SNP is "polar-left", according to the classification in Ref. [15]. Note that it is only necessary to reproduce well the functional form of $d(q)$ in the region $0.8 < q/q_e < 1.3$ because the relevant vibrational wavefunctions ($v < 10$) vanish outside this interval. However, we stress that the transition dipole moments in Supplementary Table 3 are computed using all the grid points.

The predicted transition dipole moments d_{10} and d_{21} in Supplementary Table 3 include negative values. This implies that our model can predict the sign of the transition dipole moments, which is not accessible from infrared absorption experiments.

	Theory [Debye]	Experiment [Debye]
d_{10}	-0.390	0.389
d_{21}	-0.554	0.553
d_{20}	0.023	0.037

Supplementary Table 3. **Theoretical and experimental values of transition dipole moments for SNP.** The parameters of the dipole moment function in Eq. (S9) are $d_0 = -3.35$ a.u., $c_0 = 4.8$ a.u., $c_1 = 4.8$ a.u., $\sigma = 4.0$ a.u. and $d_p = 7.35$ a.u.

Vibrational Polariton Transition Energies. The extended Multi-level Quantum Rabi (MLQR) model proposed in Ref. [15] is given by

$$\hat{H} = \omega_c \hat{a}^\dagger \hat{a} + \sum_\nu \omega_\nu |\nu'\rangle\langle\nu| + \sum_{\nu,\nu'\geq\nu} g_{\nu'\nu}(|\nu'\rangle\langle\nu| + |\nu\rangle\langle\nu'|)(\hat{a}^\dagger + \hat{a}) \qquad \text{(S10)}$$

where ω_ν is the energy of the vibrational eigenstate $|\nu\rangle$, and $g_{\nu'\nu} = \mathcal{E}_0\langle\nu'|d(q)|\nu\rangle$ are state-dependent Rabi couplings. The cavity field is described by the annihilation operator $\hat{a}$, with ω_c being the cavity frequency.

In order to obtain vibrational polariton energies, we diagonalize the Hamiltonian in Eq. (S10) by considering the lowest 30 bound Morse oscillator eigenstates and the lowest 30 Fock states of the cavity mode. From the polariton spectrum we compute the transition frequencies ω_{ij} (i, j =GS, LP_1, UP_1, LP_2, MP_2, UP_2). The transition frequencies obtained are compared with the transient transmission data for the two different cavity-coupled samples in Figs. 2 (here designated Sample 1) and 4 (Sample 2) of the main text. The results are given in Supplementary Tables 4 and 5, respectively. Supplementary Fig. 7 shows the corresponding energy level diagrams.

	Theory [cm^{-1}]	In Experiment [cm^{-1}]	$|\langle j|\hat{a}|i\rangle|^2$
GS $\rightarrow$ LP_1	1894.0	1896.0	0.608
GS $\rightarrow$ UP_1	1932.0	1931.0	0.392
$LP_1 \rightarrow LP_2$	1888.0	1896.0	0.825
$LP_1 \rightarrow MP_2$	1920.0	------	0.777
$LP_1 \rightarrow UP_2$	1964.0	------	0.006
$UP_1 \rightarrow LP_2$	1849.0	------	0.008
$UP_1 \rightarrow MP_2$	1881.0	1886.0	0.306
$UP_1 \rightarrow UP_2$	1926.0	1929.0	1.077

Supplementary Table 4. Polariton transition energies for Sample 1. Computed and experimental polariton transition energies for a sample corresponding to the one described in Fig. 2 in the main text, i.e. with a Rabi splitting of 38 cm^{-1} (Experimental value = 35 cm^{-1}) and detuning $\Delta = \omega_c - \omega_0 = -4$ cm^{-1}. The last column shows the predicted transition strengths.

	Theory [cm^{-1}]	In Experiment [cm^{-1}]	$\|\langle j\|\hat{a}\|i\rangle\|^2$
GS → LP_1	1892.0	1888.0	0.602
GS → UP_1	1936.0	1934.5	0.398
LP_1 → LP_2	1886.0	1892.0	0.873
LP_1 → MP_2	1923.0	------	0.724
LP_1 → UP_2	1973.0	------	0.005
UP → LP_2	1842.0	------	0.006
UP → MP_2	1879.0	1886.0	0.332
UP → UP_2	1929.0	1932.0	1.060

Supplementary Table 5. **Polariton transition energies for Sample 2.** Computed and experimental polariton transition energies for a sample corresponding to the one described in Fig. 4 in the main text**.** Considering a Rabi splitting of 44 cm^{-1} (Experimental value = 46.5 cm^{-1}) and detuning $\Delta = \omega_c - \omega_0 = -3$ cm^{-1}. The last column shows the predicted transition strengths.

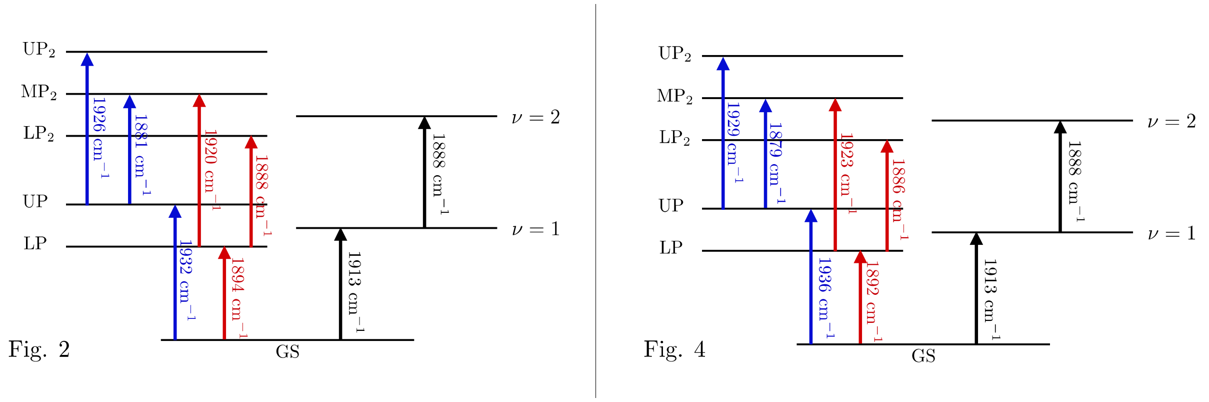

Supplementary Figure 7. Polariton energy level diagrams. Schematic polariton level diagram up to the second excited manifold for the samples described in Figs. 2 (left) and 4 (right) in the main text. Labelled vertical arrows show the dominant polariton transitions and their predicted transition frequencies in cm^{-1}. The predicted bare $v_1 \leftarrow v_0$ and $v_2 \leftarrow v_1$ vibrational frequencies are also included.

	F_{fast}	τ_{fast} (ps)	F_{slow}	τ_{slow} (ps)
		UP Pump		
UP Probe (region i)	0.57 (0.06)	0.76 (0.14)	0.43 (0.06)	5.7 (1.2)
LP Probe (region ii)	0.5 (0.2)	2.7 (0.3)	0.5 (0.2)	7 (3)
LP Probe (region iii)	0	-	1	8.4 (0.8)
		LP Pump		
LP Probe (region iv)	0.48 (0.02)	0.55 (0.04)	0.52 (0.02)	7.2 (0.6)
LP Probe (region v)	0.68 (0.03)	0.53 (0.03)	0.32 (0.03	7.6 (1.1)

Supplementary Table 6. **Kinetic fitting parameters.** Parameters determined from fits to exponential decays in Fig 5 and Supplementary Fig 8. Values determined for amplitudes (F) and decay times (τ). All data are mean ± s.d.

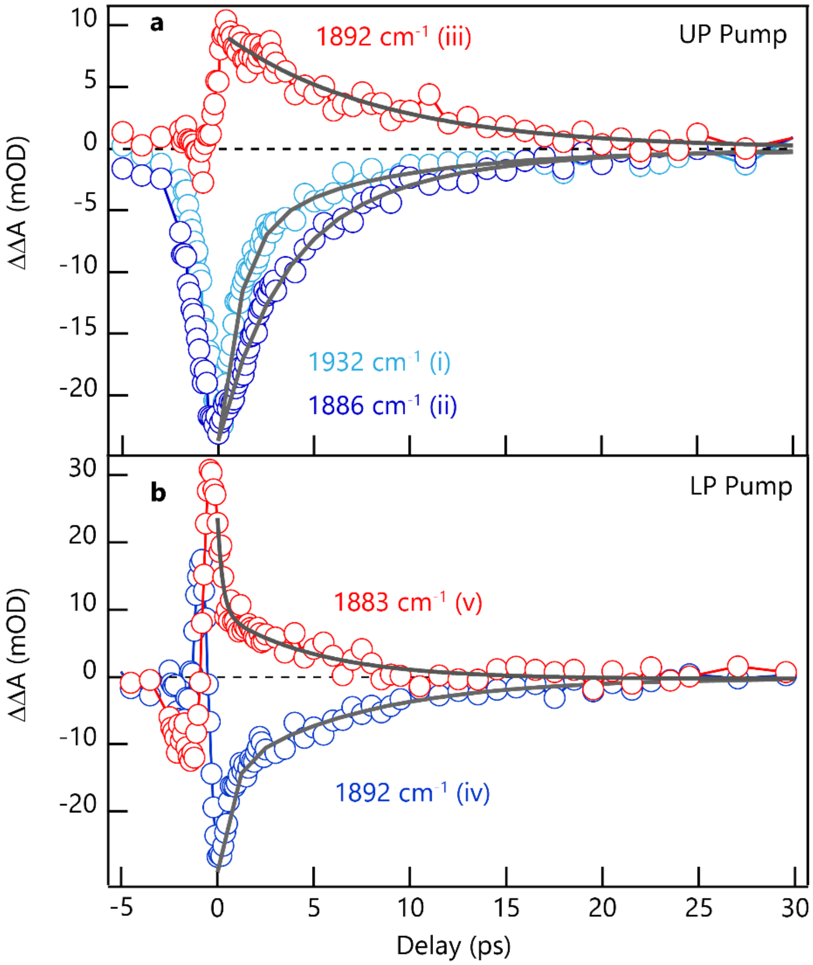

Supplementary Figure 8. Reservoir-subtracted pump-probe kinetics. Kinetics of the decay of negative and positive features that appear in the reservoir-subtracted pump-probe spectra under varying pump masks. **a.)**The red circles (iii) are data collected at ω_{probe} = 1892 cm^{-1} after excitation with the UP Pump mask. The cyan circles (i) are data collected at ω_{probe} = 1932 cm^{-1} with the UP Pump mask, i.e. the blue trace in Fig. 5 of the main text. The blue circles (ii) are data collected at 1886 cm^{-1} after excitation with the UP Pump mask. **b.)**The red circles (v) are data collected at ω_{probe} = 1883 cm^{-1} after excitation with the LP Pump mask. The blue circles (iv) are data collected at ω_{probe} = 1892 cm^{-1} with the LP Pump mask. Each gray trace is a fit of the data to a biexponential decay. The resulting fast time constant varies from 1 to 2 ps between the three datasets and the slow time constant varies from 6.5 to 8 ps for each dataset.

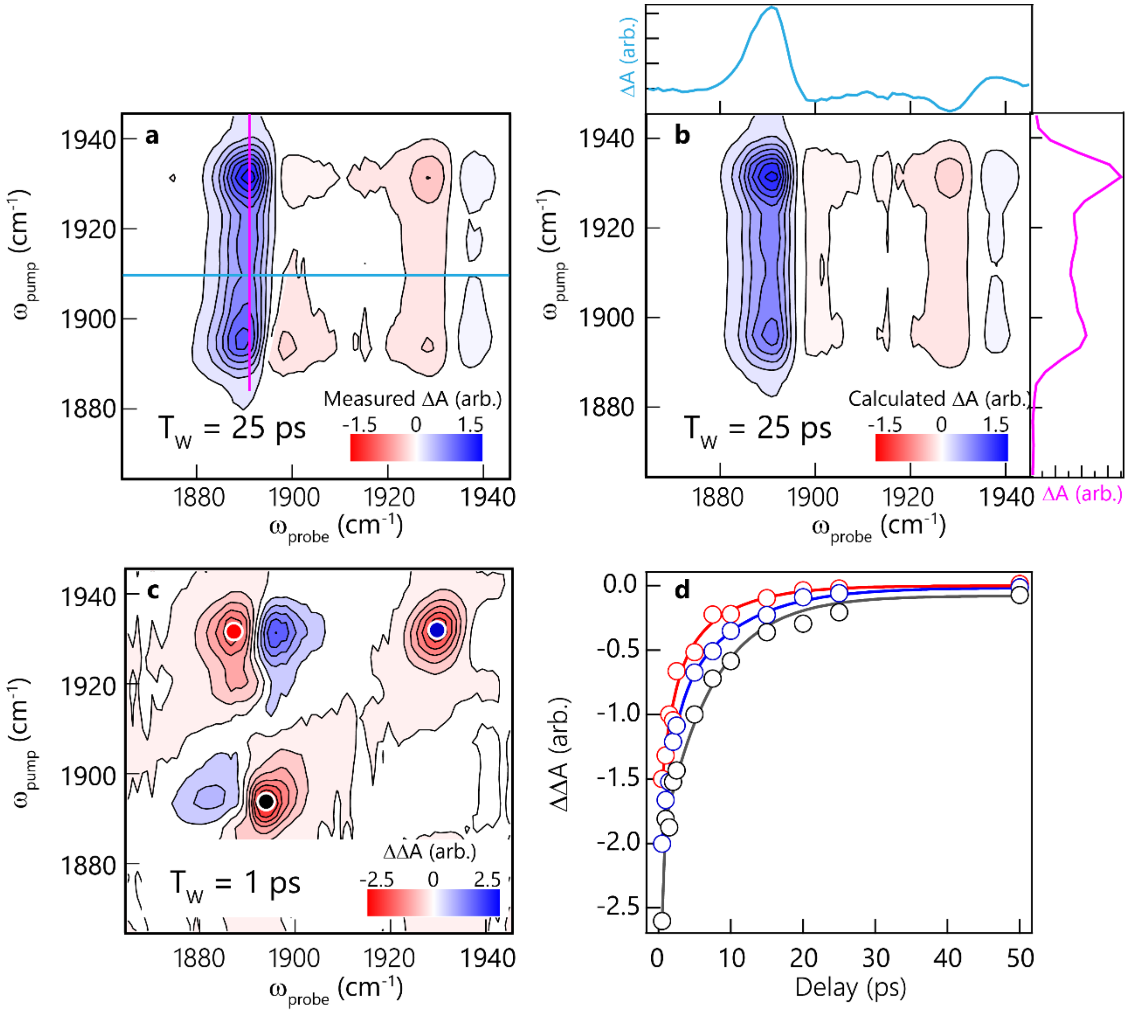

Supplementary Figure 9. 2D IR reservoir-subtracted spectra and kinetics. a) 2D IR response from cavity-coupled SNP in MeOH with T_w = 25 ps, with a response similar to that reported in the main text. **b**) Calculated reservoir response derived by multiplying the response at ω_{pump} = 1909 cm^{-1} (cyan, right sub-panel) by the response at ω_{probe} = 1892 cm^{-1} (magenta, top sub-panel). **c**) Reservoir-subtracted 2D IR spectrum at T_w = 1 ps. **d**) Time evolution of the reservoir-subtracted 2D IR response, with the red, blue, and black circles corresponding to the signal level measured at the corresponding pump and probe frequencies identified by colored circles in **c**. Each solid trace is a fit of the data to a biexponential decay. Resulting in fast time constant on the order of 1 ps and a slow component that averages 7.6 ± 1.0 ps across the three negative features in this dataset.

Supplementary Note 3. 2D IR Reservoir Subtraction.

Subtracting the reservoir-only response from 2D IR spectra is less straightforward than for pump-probe spectra because of the increased dimensionality. One must account for the pump frequency dependence of the 2D IR response. Our approach is to use the 2D IR spectrum obtained at T_w = 25 ps as representative of “late-time” response, just as we considered a 25 ps delay for the pump-probe subtraction, in order to isolate the reservoir-only response. We extract a slice of the 2D IR spectrum (Supplementary Fig. 9a) along ω_{pump} = 1909 cm^{-1}, the peak of the free-space absorption spectrum, to obtain the cyan trace

in Supplementary Fig.9b. This trace represents the probe spectrum after exciting only the reservoir. We then extract a slice of the 2D IR spectrum along ω_{probe} = 1892 cm^{-1}, where the 2D IR signal is maximized, to obtain the pink trace in Supplementary Fig. 9b. This trace represents the relative efficiency with which a given pump frequency generates the reservoir-only response. Multiplying these two traces results in a matrix plotted as contours in Supplementary Fig. 9b which corresponds to the reservoir-only 2D IR spectrum. For each T_w, we scale this matrix by the ratio of the intensity at ω_{pump} = 1909 cm^{-1}, ω_{probe} = 1892 cm^{-1}. The scaling factors we obtain decay exponentially with a 24 ps lifetime, just as we would expect from the reservoir decays in the main text. We observe no indication of modified reservoir kinetics that might indicate energy transfer from the reservoir to either polariton mode. Finally, we subtract each scaled reservoir 2D IR spectrum from the 2D IR spectrum at each T_w. The subtracted spectrum at T_w = 1 ps (Supplementary Fig. 9c) exhibits derivative-like features in the LP Probe region for both UP Pump and LP Pump. The UP probe region shows a strong negative feature for UP Pump, but no response for LP Pump. These features are in reasonable qualitative agreement with the subtracted pump-probe spectra in the main text.

We hesitate to draw strong conclusions from the subtracted 2D IR spectra because of the many assumptions that underlie the subtraction of the reservoir contribution, but identify two salient points. First, the subtracted features are not strongly stretched along the diagonal axis. We infer that, within a given pump region, the probed response is insensitive to the specific pump frequency. Put differently, there appears to be little frequency-frequency correlation in the subtracted responses. Second, the intensity of the negative features decays with similar kinetics to those obtained with pump-probe spectroscopy (Supplementary Fig. 9d). The relative paucity of datasets and time delays from the 2D IR preclude a detailed statistical analysis, but each trace yields a fast component on the order of 1 ps and a slow component that averages 7.6 ± 1.0 ps across the three features in this dataset. This similarity suggests that the presence of pump photons at both LP and UP frequencies does not dramatically change the decay pathways available to the excited system.

Supplementary References

1 Ahn, W., Vurgaftman, I., Dunkelberger, A. D., Owrutsky, J. C. & Simpkins, B. S. Vibrational strong coupling controlled by spatial distribution of molecules within the optical cavity. *ACS Photonics* **5**, 158-166 (2018).

2 Dunkelberger, A. D., Davidson, R. B., Ahn, W., Simpkins, B. S. & Owrutsky, J. C. Ultrafast transmission modulation and recovery via vibrational strong coupling. *J. Phys. Chem A* **122**, 965-971 (2018).

3 Dunkelberger, A. D. *et al.* Saturable absorption in solution-phase and cavity-coupled tungsten hexacarbonyl. *ACS Photonics* **6**, 2719-2725 (2019).

4 Dunkelberger, A. D., Spann, B. T., Fears, K. P., Simpkins, B. S. & Owrutsky, J. C. Modified relaxation dynamics and coherent energy exchange in coupled vibration-cavity polaritons. *Nat. Comm.* **7**, 13504 (2016).

5 Ribeiro, R. F. *et al.* Theory for nonlinear spectroscopy of vibrational polaritons. *J. Phys. Chem. Lett.* **9**, 3766-3771 (2018).

6 Simpkins, B. S. *et al.* Spanning strong to weak normal mode coupling between vibrational and Fabry-Perot cavity modes through tuning of vibrational absorption strength. *ACS Photonics* **2**, 1460-1467 (2015).

7 Xiang, B. *et al.* Two-dimensional infrared spectroscopy of vibrational polaritons. *PNAS* **115**, 4845-4850 (2018).

8 Long, J. P. & Simpkins, B. S. Coherent coupling between a molecular vibration and Fabry–Perot optical cavity to give hybridized states in the strong coupling limit. *ACS Photonics* **2**, 130-136 (2015).

9 Sando, G. M., Zhong, Q. & Owrutsky, J. C. Vibrational and rotational dynamics of cyanoferretes in solution. *J. Chem. Phys.* **121**, 2158-2168 (2004).

10 Brookes, J. F., Slenkamp, K. M., Lynch, M. S. & Khalil, M. Effect of solvent polarity on the vibrational dephasing dynamics of the nitrosyl stretch in the Fe^{II} complex revealed by 2D IR spectroscopy. *J. Phys. Chem A* **117**, 6234-6243 (2013).

11 Smith, M. H., Rinsland, C. P., Fridovich, B. & Rao, K. N. *Intensities and Collision Broadening Parameters from Infrared Spectra Ch. 3* in *Molecular Spectroscopy: Modern Research* (eds K. N. Rao & C. W. Mathews) 111-248 (Academic Press, New York and London, 1985).

12 Keim, E. R., Polak, M. L., Owrutsky, J. C., Coe, J. V. & Saykally, R. J. Absolute infrared vibrational band intensities of molecular ions determined by direct laser absorption spectroscopy in fast ion beams. *J. Chem. Phys.* **93**, 3111-3119 (1990).

13 Yanai, T., Tew, D. & Handy, N. A new hybrid exchange-correlation functional using the Coulomb-attenuating method (CAM-B3LYP). *Chem. Phys. Lett.* **393**, 51-57 (2004).

14 Marston, C. C. & Balint-Kurti, G. G. The Fourier grid Hamiltonian method for bound state eigenvalues and eigenfunctions. *J. Chem. Phys.* **91**, 3571-3576 (1989).

15 Hernandez, F. J. & Herrera, F. Multi-level quantum rabi model for anharmonic vibrational polaritons. *J. Chem. Phys.* **151**, 144116 (2019).

16 Triana, J. F., Hernandez, F. J. & Herrera, F. The shape of electric dipole function determines the sub-picosecond dynamics of anharmonic vibrational polaritons. *J. Chem. Phys.* **152**, 234111 (2020).
